# Supplementary material for: Phosphoethanolamine Transferases as Drug Discovery Targets for Therapeutic Treatment of Multi-Drug Resistant Pathogenic Gram-Negative Bacteria
Source: Antibiotics (Basel). 2023 Aug 29;12(9):1382. doi: 10.3390/antibiotics12091382 (PMC10525099; doi:10.3390/antibiotics12091382)
Supplement: Supplementary file 1 [file antibiotics-12-01382-s001.zip › antibiotics-2569363-supplementary.pdf]

Table S1: The representative PEA transferases for constructing phylogenetic tree.

Origin	Strain	Accession number	Protein name	Reference
Escherichia coli	K12	AAC77075	EptA	[17]
Salmonella enterica Typhimurium	LT2	P36555	EptA	[34]
Acinetobacter baumannii	ATCC 17978	G1CBN7	EptA	[36]
Shigella flexneri	301	A0A0H2VXP0	EptA	[17]
Klebsiella_pneumoniae	HS11286	A0A0H3GLS1	EptA	[17]
Helicobacter pylori	26695	ABE02822	EptA	[38]
Pseudomonas aeruginosa	PAO1	Q9HYT4	EptA	[17]
Vibrio cholerae	O1 biovar El Tor N16961	Q9KKK2	EptA	[41]
Neisseria meningitidis	MC58	Q7DD94	EptA	[49]
Neisseria gonorrhoeae	FA1090	KAE9493802	EptA	[17]
Haemophilus ducreyi	35000HP	AAP95743	EptA	[53]
Cronobacter sakazakii	ATCC BAA-894	A7MFE0	ESA_RS09200	[54]
Vibrio parahaemolyticus	RIMD 2210633	VP_RS21300	VP_RS21300	[55]
Pasteurella multocida	AL435	OBP27748	PetL	[18]
Moraxella catarrhalis	BC1	EGE18576.1	ICR	[57]
Moraxella osloensis	CCUG 350	AME01623	ICR	[15]
Escherichia coli	GDP6F1	ATZ71845	MCR-1	[71]
Escherichia coli	KP37	SBV31106	MCR-2	[72]
Escherichia coli	WJ1	WP_104769708	MCR-3	[58]
Salmonella enterica Typhimurium	R3445	ASR73329	MCR-4	[59]

Salmonella enterica Typhimurium	B *d*Ta+ isolate 13-SA01718	WP_229654935	MCR-5	[60]
Moraxella pluranimalium	AM884564	AVA17336	MCR-6	[73]
Klebsiella pneumoniae	SC20141012	WP_104009851	MCR-7	[62]
Klebsiella pneumoniae	KP91	UIX51983	MCR-8	[74]
Salmonella enterica Typhimurium	HUM_TYPH_WA_10_R9_3274	QCC62183	MCR-9	[65]
Enterobacter roggenkampii	WCHER090065	QDO66747	MCR-10	[64]
Pasteurella multocida	AL435	OBP26495	PetK	[18]
Escherichia coli	W3110	Q38J80	EptB	[81]
Salmonella enterica Typhimurium	ATCC14028	UVD93218	EptB	[86]
Yersinia pestis	subsp. Pestis bv. Medievalis	A0A5P8RYF1	EptB	[88]
Campylobacter jejuni	NCTC 11168	Q0PBP9	EptC	[78]
Escherichia coli	K12	P0CB39	EptC	[21]
Salmonella enterica Typhimurium	LT2	Q7CPC0	EptC (CptA)	[22]
Neisseria gonorrhoeae	FA1090	WP_171000415	Lpt3	[99]
Neisseria meningitidis	MC58	WP_118818077	Lpt3	[92]
Pasteurella multocida	P1702	D0EAD9	Lpt3	[56]
Neisseria meningitidis	MC58	C5IWX1	Lpt6	[92]
Neisseria gonorrhoeae	FA19	WP_050162946	Lpt6	[93]
Haemophilus influenzae	Rd	WP_233775356	Lpt6	[91]
Proteus mirabilis	HI4320	B4F0R8	EptC	[95]
Pasteurella multocida	AL435	OBP28286	PetG	[18]
Shigella flexneri	2002017	ADA77030	Lpt-O	[24]

Neisseria gonorrhoeae	FA19	Q9RMJ3	PptA	[30]
Neisseria meningitidis	Z2491	WP_002237381	PptA	[29]
Salmonella enterica Typhimurium	LT2	Q7CPI7	BcsG	[27]
Escherichia coli	K12	P37659	BcsG	[25]
Escherichia coli	K12	P75785	OpgE	[28]
Haemophilus ducreyi	35000HP	AAP95341	PtdA	[53]
Haemophilus ducreyi	35000HP	AAP96378	PtdB	[53]
